# Supplementary material for: Health workers’ perception of malaria rapid diagnostic test and factors influencing compliance with test results in Ebonyi state, Nigeria
Source: PLoS One. 2019 Oct 17;14(10):e0223869. doi: 10.1371/journal.pone.0223869 (PMC6797183; doi:10.1371/journal.pone.0223869)
Supplement: S1 Questionnaire — (DOCX) [file pone.0223869.s001.docx]

**Informed Consent Form**

**Introduction**

My name is **OBI, Izuchukwu Frank.** I am a Postgraduate student (MPH Field Epidemiology) in the Department of Community Medicine, Ahmadu Bello University Zaria and also a resident of the Nigeria Field Epidemiology and Laboratory Training Program. I am conducting a research titled: **PERCEPTION OF MALARIA RAPID DIAGNOSTIC TEST AND FACTORS INFLUENCING COMPLIANCE WITH TEST RESULT AMONG HEALTH WORKERS IN EBONYI STATE.** To carry out this study, I will be asking you and other 300 respondents like you a number of questions which will take about 20 minutes to complete. Please note that all information collected through this questionnaire will be kept confidential.

The outcome of this research will be useful in understanding what health workers in Ebonyi State think about Malaria Rapid Diagnostic Test and whether they comply with the test results during patient treatment. There is no risk in taking part in this survey and you have the right to decline participation at any time. Your name will NOT be captured on the forms I use to write down your response to questions or the report of this study. Your participation in this research is entirely voluntary. Please make sure any doubts you have are cleared before you agree to take part.

**Consent to Participate**

I have read the description of the research and also talked it over with the Researcher to my satisfaction. I understand that my participation is voluntary.

I know enough about the purpose, methods, and benefit attached to the research study to judge that I want to take part in it. I understand that I may freely stop being part of this study at any time.

Name or Initials of Respondent. ……………………………………………………………….

Signature....................................... Date.................................

Name of Interviewer. ………………………………………………………………..

Signature ...................................... Date**..................................** Questionnaire ID..................

This research has been approved by the Ethics Committee of Ebonyi State Ministry of Health and the Chairman of this Committee can be reached on **+234803997766**.

In addition, if you have any question about your participation in this research, you can contact the Principal Investigator, **Dr. OBI, Izuchukwu Frank**, Department of Community Medicine, Ahmadu Bello University Zaria. Phone: **+2348064446077** Email: **obifrank03@gmail.com**

**QUESTIONNAIRE**

**Introduction**

Good day Sir/ Madam,

I am **OBI, Izuchukwu Frank.** A Postgraduate student (MPH Field Epidemiology) in the Department of Community Medicine, Ahmadu Bello University Zaria and also a resident of the Nigeria Field Epidemiology and Laboratory Training Program. I am conducting a research titled: **PERCEPTION OF MALARIA RAPID DIAGNOSTIC TEST (mRDT) AND FACTORS INFLUENCING COMPLIANCE WITH TEST RESULT AMONG HEALTH WORKERS IN EBONYI STATE.** I wish to appeal for your cooperation to participate in this study by objectively answering the questions from the Research Assistants. Participation in this study is voluntary; one can withdraw from the study at anytime without any penalty whatsoever. The information and results of your responses will be treated as confidential and strictly for academic purposes only and your name will not be written down anywhere in the questionnaire.

Thank you.

Do you agree to the interview? Yes No

Date of Interview _______________________________

Name of interviewer ______________________________________

Questionnaire ID ______________

Name of Facility ______________________________________

Type of Health Facility: Public Private

Level of Health Facility: Primary Secondary Tertiary

Location of Health Facility: Urban Rural

Local Government Area

Senatorial District

**SECTION A: SOCIO-DEMOGRAPHIC CHARACTERISTICS**

1. Age as at last Birthday (in years) ___________
2. Sex: Male Female
3. Professional Cadre: Junior CHEW CHEW Pharmacy Tech

EHO Auxiliary Nurse Science Lab Tech Med Lab Scientist

CHO Registered Nurse Pharmacist Medical Doctor

Others (specify) _________________

1. What is your highest educational qualification (BMLS, MBBS, FWACP etc.)? ___________
2. How many **years** have you worked as a Health professional? _________________

**SECTION B: MALARIA DIAGNOSIS IN FACILITY AND TRAINING**

1. What test do you use to diagnose malaria in this facility? a. No test done b. mRDT only

c. mRDT and Microscopy d. Microscopy alone e. Others (specify) _____________

1. Do you currently have mRDT kits in this facility? Yes No
2. Do patients pay for mRDT in this facility? Yes No

If Yes to 6 above, how much do they pay? _________________

1. At what point during patient care do you conduct mRDT?
   1. Before consultation, while still at the waiting area
   2. At the point of consultation inside the consulting room
   3. After consultation patient is sent to the lab for mRDT
2. Do you wait for mRDT result to become available before prescribing antimalarial drug to febrile patients? Always Most times Sometimes I don’t wait
3. Who performs mRDT in your facility? (Tick all that apply, multiple selections allowed)
   1. A dedicated staff outside the consulting room
   2. A dedicated staff inside the consulting room
   3. Whoever is attending to the febrile patient performs the test at the point of care
   4. Laboratory outside the facility
   5. Others (specify)
4. Since you started treating febrile patients, have you been trained on malaria case management? Yes No

*(If Yes to 12, go to the next question, if No, jump to question 17)*

1. When was the last training done? (Approximate time to the nearest option and select one)?
   1. Less than 1 year ago b. About 1 year ago c. About 2 years ago

d. About 3 years ago e. About 4 years ago f. ≥5 years ago

1. Who conducted the last training you attended on malaria case management?
2. Federal Ministry of Health
3. Ebonyi State Malaria Elimination Program
4. Partner organization (e.g. WHO, UNICEF, MAPs)
5. Indicate the nature of malaria related trainings you have attended(multiple selections allowed)
   1. Training on malaria case management without mRDT training
   2. Training on malaria case management inclusive of RDT training
   3. Stand alone training on mRDT with practical/ hands on sessions
   4. Stand alone training on mRDT without practical/ hands on sessions
   5. Others (specify) _________________
6. How long did the last training last (How many days)? __________________________
7. Since you started treating patients, have you been trained on other causes of fever apart from malaria? Yes No
8. Since you started treating patients, have you been trained on Health worker-Client negotiation skills? Yes No

**SECTION C: PERCEPTION OF MALARIA RDT**

*Select (√) your level of agreement/ disagreement with the following statements:*

| **S/N** | **Statement** | **Strongly Disagree** | **Disagree** | **Neutral** | **Agree** | **Strongly Agree** |
| --- | --- | --- | --- | --- | --- | --- |
| 19. | Use of clinical symptoms and signs **alone** is an accurate way of confirming malaria diagnosis |  |  |  |  |  |
| 20. | It is important to **always** confirm malaria diagnosis with lab test before treatment |  |  |  |  |  |
| 21. | Use of mRDT is an effective way of confirming malaria diagnosis |  |  |  |  |  |
| 22. | Microscopy is more effective than mRDT in the diagnosis of malaria |  |  |  |  |  |
| 23. | Malaria-RDT is more effective than microscopy in the diagnosis of malaria |  |  |  |  |  |
| 24. | Microscopy and mRDT are **equally** effective in the diagnosis of malaria |  |  |  |  |  |
| 25. | I fully trust a **positive** mRDT result as being truely positive |  |  |  |  |  |
| 26. | I fully trust a **negative** mRDT result as being truely negative |  |  |  |  |  |
| 27. | I am more confident in positive mRDT result than in a negative one |  |  |  |  |  |
| 28. | I am more confident in a negative mRDT result than in a positive one |  |  |  |  |  |
| 29. | I am equally confident in a positive and a negative mRDT result |  |  |  |  |  |
| 30. | I have no confidence in both a positive and a negative mRDT result |  |  |  |  |  |
|  | **I will consider prescribing antimalarial drugs to a patient with negative mRDT result under the following conditions:** | **Strongly Disagree** | **Disagree** | **Neutral** | **Agree** | **Strongly Agree** |
| 31. | If I have strong clinical suspicion of malaria |  |  |  |  |  |
| 32. | If patient reported taking antimalarial drug before presentation to me |  |  |  |  |  |
| 33. | If I suspect that patient has a low plasmodium parasite level not detected by mRDT |  |  |  |  |  |
| 34. | If patient pressurizes me to prescribe for him/her |  |  |  |  |  |
| 35. | If I don’t trust the mRDT I am using |  |  |  |  |  |
| 36. | If I don’t trust my skills in performing mRDT |  |  |  |  |  |

**SECTION D: COMPLIANCE WITH MALARIA RAPID DIAGNOSTIC TEST RESULT**

1. What do you usually do for patients with negative mRDT result? *(select one)*
   1. I give them antimalarial drugs
   2. I give analgesics and ask them to come back if fever persists
   3. I ask them to come for repeat mRDT in the next 24-48 hours
   4. I give them Antibiotics
   5. I send them for microscopy
   6. I send them for other lab investigations
   7. I refer them to a doctor
   8. I don’t know what else to do
   9. Others (specify)
2. Have you ever prescribed antimalarial drug for a patient with negative mRDT result?

Yes No I can’t remember

*(If yes, go to the next question, if no, jump to question 47)*

1. In the past 6 months, have you prescribed antimalarial drugs for a patient with negative mRDT result? Yes No I can’t remember
2. If yes to 39, approximately how many times did you prescribe antimalarial drug for a patient with negative mRDT result in the past 6 months?
   1. 1-5 times b. 6-10 times c. 11-15 times d. 16-20 times e. >20 times
3. When was the last time you prescribed antimalarial drug to a patient with negative mRDT?
   1. Within the past seven days b. Within the past 14 days c. Within the past 28 days

d. Within the past three months e. Within the past six months

f. More than six months ago

1. Which antimalarial drug(s) did you prescribe for the last patient in question 41 above?
   1. ACT b. Quinine c. Chloroquine d. Sulfadoxine-Pyrimethamine

e. Others (specify)

1. Why did you prescribe antimalarial drugs to these patients with negative mRDT results?
   1. I strongly suspected malaria clinically
   2. Patient reported taking antimalarial drug before presenting to me
   3. I believed patient had low parasite level that could not be detected by the mRDT
   4. Patient pressurized me to prescribe
   5. I did not trust the brand of mRDT I was using
   6. I believed they were falsely classified as negative by MRDT
   7. I did not know what else to treat for when they tested negative to MRDT
   8. Others (specify)
2. Did you prescribe any other drug(s) for the patient in Question 41 above? Yes No
3. Which other drug(s) did you prescribe for the patient alluded to above? *(multiple selection )*
   1. Paracetamol b. Blood tonics c. Antibiotics d. Antiemetics

e. Others (specify)

1. What was the outcome of the treatment you gave to the patient(s) alluded to above?
   1. They got better b. They got worse c. The died

d. I referred them to another facility e. I can’t remember

**SECTION D: SUPERVISION AND AVAILABILITY OF JOB AIDS**

1. Have you ever received supportive supervision on case management of malaria?

Yes No

*If Yes to question 47, continue with 48; if No, jump to question 53.*

1. In the past 6 months, have you received supportive supervision on malaria case management?

Yes No

1. Who conducted the last supportive supervision?
2. A senior officer from this facility
3. A senior officer from the Local Government
4. An officer from the State Ministry of Health ( e.g. SMEP)
5. An officer from the Federal Ministry of Health
6. An officer from Development Partners *(WHO, MAPS...) Specify* __________________
7. Did the supervisor use a checklist? Yes No
8. Did the supervisor give you feedback ? Yes No

1. If yes to 51, what type of feedback? Verbal Written Both verbal and written

1. Do you have mRDT User’s Manual in your facility? Yes No
2. Does your facility have any algorithm/guide showing what should be done for a febrile patient with negative mRDT result? Yes No

1. If yes to 54, is the algorithm displayed clearly at the point of care? Yes No

1. Is any form of quality control checks done on your mRDTs in this facility? Yes No
2. If yes to 56, what form of quality control check is done?
   1. Monitoring of mRDT procedures by superiors
   2. Monitoring of mRDT storage by superiors
   3. Periodic validation of mRDT results with Microscopy
   4. Others (specify)
3. Who conducts the mRDT quality control checks *(SMEP, MAPS etc.)*?
   1. Superiors from my facility
   2. Quality control officers from the LGA
   3. Quality control officers from the State Malaria Elimination Program
   4. Quality control officers from the Federal Ministry of Health
   5. Quality control officers from Partner Organisations *(MAPS, WHO etc.)*
   6. Others (specify)
4. How often are the Quality control checks done *(3 monthly, 6 monthly etc.)*?

1. Do you receive feedbacks on the mRDT quality control results? Yes No

**THANK YOU**
